# Whole genome comparisons of *Fragaria*, *Prunus *and *Malus *reveal different modes of evolution between Rosaceous subfamilies

**DOI:** 10.1186/1471-2164-13-129

**Published:** 2012-04-04

**Authors:** Sook Jung, Alessandro Cestaro, Michela Troggio, Dorrie Main, Ping Zheng, Ilhyung Cho, Kevin M Folta, Bryon Sosinski, Albert Abbott, Jean-Marc Celton, Pere Arús, Vladimir Shulaev, Ignazio Verde, Michele Morgante, Daniel Rokhsar, Riccardo Velasco, Daniel James Sargent

**Affiliations:** 1 Department of Horticulture and Landscape Architecture, Washington State University, Pullman, WA 99164, USA; 2 Istituto Agrario San Michele all'Adige, Via E. Mach 1, 38010 San Michele all'Adige, Italy; 3 Computer Science, Saginaw Valley State University, University Center, MI 48710, USA; 4 Horticultural Sciences Department, University of Florida, Gainesville, Florida 32611, USA; 5 Department of Horticultural Science, North Carolina State University, Campus Box 7609, Raleigh, NC 27695, USA; 6 Department of Genetics and Biochemistry, Clemson University, Clemson, SC 29634, USA; 7 UMR Génétique et Horticulture (GenHort), INRA/Agrocampus-ouest/Université d'Angers, Centre Angers-Nantes, 42 rue Georges Morel -, BP 60057, 49071 Beaucouzé cedex, France; 8 IRTA, Centre de Recerca en Agrigenòmica CSIC-IRTA-UAB-UB, Campus UAB, Bellaterra (Cerdanyola del Vallès), 08193 Barcelona, Spain; 9 Department of Biological Sciences, University of North Texas, 1155 Union Circle, Denton, Texas, USA; 10 CRA - Fruit Tree Research Center, Via di Fioranello, 52, 00134 Rome, Italy; 11 Istituto di Genomica Applicata, Parco Scientifico e Tecnologico L. Danieli, via Linussio, 51, 33100 Udine, Italy; 12 DOE Joint Genomics Institute, 2800 Mitchell Dr, Walnut Creek, CA, USA

**Keywords:** Rosaceae, Comparative genomics, Evolution

## Abstract

**Background:**

Rosaceae include numerous economically important and morphologically diverse species. Comparative mapping between the member species in Rosaceae have indicated some level of synteny. Recently the whole genome of three crop species, peach, apple and strawberry, which belong to different genera of the Rosaceae family, have been sequenced, allowing in-depth comparison of these genomes.

**Results:**

Our analysis using the whole genome sequences of peach, apple and strawberry identified 1399 orthologous regions between the three genomes, with a mean length of around 100 kb. Each peach chromosome showed major orthology mostly to one strawberry chromosome, but to more than two apple chromosomes, suggesting that the apple genome went through more chromosomal fissions in addition to the whole genome duplication after the divergence of the three genera. However, the distribution of contiguous ancestral regions, identified using the multiple genome rearrangements and ancestors (MGRA) algorithm, suggested that the *Fragaria *genome went through a greater number of small scale rearrangements compared to the other genomes since they diverged from a common ancestor. Using the contiguous ancestral regions, we reconstructed a hypothetical ancestral genome for the Rosaceae 7 composed of nine chromosomes and propose the evolutionary steps from the ancestral genome to the extant *Fragaria*, *Prunus *and *Malus *genomes.

**Conclusion:**

Our analysis shows that different modes of evolution may have played major roles in different subfamilies of Rosaceae. The hypothetical ancestral genome of Rosaceae and the evolutionary steps that lead to three different lineages of Rosaceae will facilitate our understanding of plant genome evolution as well as have a practical impact on knowledge transfer among member species of Rosaceae.

## Background

The Rosaceae is one of the most economically important and morphologically diverse plant families with over 90 genera containing more than 3000 species. The family contains three sub-families; the Dryadoideae, the Rosoideae and the Spireaeoideae, with the economically-important genera *Prunus *and *Malus *contained within the Spireaeoideae, whilst *Fragaria *is a member of the Rosoideae [1]. The base chromosome number of the many genera within the family ranges from *x *= 7 to *x *= 17, and recent research has suggested that the ancestral chromosome number for Rosaceae may have been *x *= 9 [2,3]. As in many other plant families, comparative genomics will enhance our understanding of genome structure and function and the evolutionary forces that have led to the current chromosomal configurations of the numerous Rosaceous species, and in turn to the mechanisms responsible for the wealth of morphological diversity encompassed by the family. An understanding of the degree of conservation of genome structure and function between related genera will enable inferences to be made about the genomic positions of genes controlling common traits among genera and permit information gained in one species to inform investigations in another.

The recent availability of whole genome sequences has permitted the delineation of syntenic blocks at high resolution and from this the evolutionary history in plant lineages can be inferred. In the grasses, paleogenomic modeling, using sequences of the maize, rice, and sorghum genomes as well as large sets of genetically mapped genes in wheat and barley, led to the proposal of an ancestral grass karyotype for the five ancestral chromosomes [4,5] from which all modern grass genomes evolved. The recent sequencing of the *Brachypodium *genome [6] revealed a whole-genome paleo-duplication in *Brachypodium *chromosomes, whilst comparisons of the *Brachypodium*, rice and sorghum genome sequences revealed orthologous relationships that were consistent with the evolution of the extant *Brachypodium *genome from an ancestral genome containing five chromosomes.

Similarly, in the dicots, whole genome sequencing has revealed patterns of genome evolution that it had not been possible to detect using comparative mapping of orthologous markers. The sequencing of the grapevine genome [7] and its comparison to the genomes of *Arabidopsis *and poplar permitted the identification of a paleo-hexaploidisation event in the common lineage of the three species which occurred after the monocotyledonous and dicotyledenous plant lineages diverged. This hexaploidisation event had not previously been identified, despite the whole genome sequences of *Arabidopsis *and poplar being available for some time [8,9]. This was primarily due to the subsequent polyploidisation events that had occurred in the genomes of these species (once in the case of poplar, and twice in the case of *Arabidopsis*) since they diverged from a common ancestor. Thus, analyses based on higher levels of resolution, particularly those based on whole genome sequence data, reveal evermore complex patterns of genome evolution between species, but at the same time provide compelling evidence to support models of genome evolution and deduced ancestral chromosomal configurations.

So far no studies have been performed that have compared whole genome sequences of plant species that belong to different genera of the same family. In Rosaceae, as well as in other economically important plant families including Poaceae, Solanaceae, Brassicaceae and Fabaceae [10-14], the comparative genomics studies have been performed using conserved genetic markers. Dirlewanger et al [15] first identified high levels of conservation of marker presence and order between three of the eight linkage groups of the *Prunus *reference map [16], and seven of the 17 linkage groups of the apple map [17], demonstrating that markers mapping to a single *Prunus *linkage group were located on two homeologous linkage groups on the *Malus *linkage map and that large conserved syntenic blocks were clearly identifiable within the two genera. A number of other studies were also performed using PCR-based markers that had been developed from both *Malus *and *Fragaria*, which were applied to comparative mapping between *Prunus *and these other members of the Rosaceae [18,19]. High level of co-linearity within the sub-family Maloideae between the genomes of *Malus *and *Pyrus *has also shown by comparative mapping using simple sequence repeat (SSR) markers [20]. Vilanova et al [2] reported a genome-wide inter-generic comparison of genetically mapped orthologous markers between diploid *Fragaria *and *Prunus *showing sufficiently well conserved macro-synteny to enable the reconstruction of a hypothetical ancestral genome for Rosaceae containing nine chromosomes. The study however also revealed a number of large-scale chromosomal rearrangements, including translocations of large syntenic blocks and numerous fusion-fission events that had occurred in the evolutionary history of the two genera. More recently, using the whole genome sequence from the apple cultivar 'Golden Delicious' [21] and sequence data from 1,473 markers mapped in *Prunus *and *Fragaria*, including Rosaceous conserved orthologous sequences (RosCOS) [22], Illa et al [3] performed a genome-wide comparison between all three genera. Analyses based on the positions of the 129 markers revealed clear, conserved, syntenic blocks that were common to all three genomes, with a single syntenic block in *Prunus *corresponding to one or two syntenic regions in *Fragaria*, and two or four syntenic regions in apple. Illa et al [3] reconstructed a hypothetical ancestral genome for the Rosaceae containing nine chromosomes (*x *= 9), consistent with the report of Vilanova et al [2]. The data suggested that the resolution of studies based on modest numbers of markers was perhaps not sufficient to elucidate the true number of small scale genomic inversions that have taken place in genome evolution within the Rosaceae, which may have played an important role in speciation within the family. Thus, an evaluation of the conservation of synteny between *Fragaria*, *Malus *and *Prunus *based on whole genome sequence data may reveal much about sequence evolution in this closely-related, yet morphologically diverse family that has been hitherto undetected.

The genomes of three Rosaceous genera of significant economic importance, *Fragaria *[23], *Malus *[21] and *Prunus *[24] have recently been sequenced, presenting an exciting opportunity for high-resolution genome comparison. Here we report results from comparison of whole genome sequences of the three species of Rosaceae and the genome of *Vitis vinifera*, included as an outgroup species representing a basal rosid genome. We were able to identify the orthologous regions among the three Rosaceous species at a much higher-resolution than has previously been reported. This higher-resolution enabled us to detect different patterns of genome evolution between the sub-families of Rosaceae. Furthermore, we reconstructed a hypothetical Rosaceae ancestral genome using the Multiple Genome Rearrangements and Ancestors (MGRA) algorithm and further manual analyses.

## Results and Discussion

### Evaluation of orthologous regions between taxon pairs

The RosCOS markers used previously by [3] are a useful resource in comparative genome alignment and as such revealed insights into the patterns of genome evolution on a macro-syntenic scale in that study. Since the RosCOS are an important resource for future comparative studies, we anchored them to the orthologous regions (ORs) identified in this investigation (Additional file 3: Table S1). However, since orthologous genes in two species do not necessarily reside in large orthologous regions of the genome, using a relatively small set of orthologous sequences (as in the case of the RosCOS markers) in the detection of microsynteny would only be possible in genomic regions where the order of a large number of orthologs is conserved among related genomes. With only 800 mapped RosCOS available for study, it was difficult to detect orthologous regions at very high levels of resolution. Capitalising on the availability of whole genome sequences with many more predicted genes (27,243 in peach, 33,264 in strawberry and 43,335 in the primary assembly of apple), along with Mercator [24], which selects one to one orthologous regions based on the large numbers of exons available for study, meant that we were able to detect the conservation of synteny between the genomes at a much finer level in this investigation than in previous studies.

**Figure 1:**
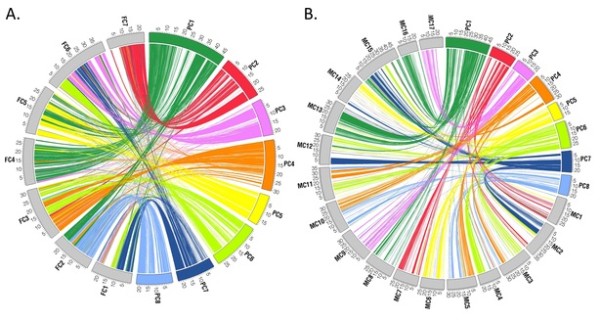
**Orthology map identified between three Rosaceous genera based on whole genome sequence analysis**. The lines link one to one orthologous regions, identified using Mercator program [25]. A. Comparison between *Prunus *and *Fragaria*, B. Comparison between *Prunus *and *Malus*. Data were plotted using Circos [42]. Colors for plots A and B follow the same pattern based on *Prunus *chromosomes.

Thus, the evolutionary history of Rosaceous genomes was investigated through the detection of ORs between *Prunus *and *Fragaria *or *Malus*, using Mercator [25]. A total of 1281 ORs were obtained in the comparison between *Prunus *and *Fragaria*, with the longest region of 1.7 Mb of PC3 and 1.4 Mb of FC6 (Table 1). The mean number of matching exons in each OR was 17 and the mean lengths of ORs were 98.8 kb in *Prunus *and 98.4 kb in *Fragaria *(Table 1). Figure 1 shows the ORs between *Prunus *and *Fragaria *(A) and *Prunus *and *Malus *(B). In most cases, each peach chromosome showed major orthology to one strawberry chromosome, but to two or more apple chromosomes, clearly indicating that the whole genome duplication (WGD) in apple occurred following the divergence of the three genera. The ortholgous relationships between chromosomes of *Fragaria *and *Prunus *were clear, with the majority of ORs on *Prunus *chromosomes PC2, PC3, PC4, PC5, and PC8 each corresponding to single homologous chromosome in *Fragaria*, FC7, FC6, FC3, FC5, and FC2, respectively. The majority of ORs on PC7 corresponded to two *Fragaria *chromosomes, FC1 and FC6, and those on PC6 corresponded to three regions of the *Fragaria *genome on FC1, FC3 and FC6. The *Prunus *ORs on PC1 were the most widely distributed within the *Fragaria *genome, with ORs corresponding to multiple homologous chromosomal regions, but with one major syntenic relationship with FC4 (Figure 1A, Table 2).

**Table 1:** Number and length of orthologous regions (ORs) in two-genome and three genome comparisons

Orthology Analysis	No. OR	Mean No. Matching Exons	Mean Length in Kb (Prunus|Fragaria|Malus)	Largest Length in Mb (Prunus|Fragaria|Malus)
Prunus and Fragaria	1281	17	98.8|98.4|NA	1.7|1.4|NA

Prunus and Malus	349	23	200.9|NA|260.5	6.1|NA|7.5

*Prunus and Malus (Split into two sub_genomes)	706	22	175.9|NA|222.9	5.5|NA|9.1

Prunus, Fragaria and Malus	1399	**27	149.4|133.5|82.4	3.5|1.3|2.6

*The *Malus *chromosomes were divided into sub-genome 1 and 2 prior to the analyses (see Materials and Methods) so that Mercator would find ORs in each *Malus *subgenome.

**Number includes the matching exons in two of the three genomes compared.

**Table 2:** Major orthologous chromosomes among *Prunus*, *Fragaria *and *Malus *

*Prunus*	*Fragaria*	*Malus*
PC1	FC2, FC4, FC5	MC13/MC16, MC8/MC15

PC2	FC7	(MC1, MC2)/MC7

PC3	FC6	MC9/MC17

PC4	FC3	MC3/MC11, MC5/MC10

PC5	FC5	MC14/MC6

PC6	FC1, FC3, FC6	MC2/MC15, MC3/MC11, MC4/MC12

PC7	FC1, FC6	MC2/MC15, M14/M12

PC8	FC2	MC5/MC10, MC3/MC11

The orthologous chromosomes were identified based on the result from orthology analysis using whole genome sequences (Figure 1).

The analysis between *Prunus *and *Malus *produced fewer, but larger ORs with a greater number of matching exons. The smaller number of ORs may reflect the fact that the primary assembly of apple does not include all the predicted genes sequenced. A total of 349 ORs were obtained, with the longest region of 6.6 Mb of PC3 and 7.5 Mb of MC9 (Table 1). The mean number of matching exons in ORs was 23 and the mean lengths of ORs were 200.9 kb in *Prunus *and 260.5 kb in *Malus *(Table 1). At the chromosome level, the analysis revealed more complex relationships between the two genera than between *Prunus and Fragaria*. ORs on PC3 and PC5 each corresponded to ORs on two major *Malus *chromosomes, MC9 and MC17, and MC6 and MC14, respectively. The two sets of *Malus *chromosomes, MC9/MC17 and MC6/MC14, were two of the chromosome doublets that contain large syntenic regions indicative of the recent WGD in *Malus *lineage which agrees with previous hypotheses that the *Malus *genome went through relatively recent Pyreae-specific WGD [3,21], that occurred following the divergence of the *Malus *and *Prunus *lineages, as no evidence of such a WGD is present in the strawberry and peach genomes [23,24]. Orthologous regions in PC2 corresponded to major ORs on three *Malus *chromosomes, MC1, MC2 and MC7. ORs on PC1, PC4, and PC7 each corresponded to ORs on four *Malus *chromosomes, whilst ORs on PC6 corresponded to ORs on multiple *Malus *chromosomes (Figure 1B, Table 2). The observation that each chromosome of *Prunus *corresponded to ORs in two or more chromosomes of *Malus*, even though Mercator detects ORs in one to one relationships, suggests both sets of chromosomes generated by WGD retained orthologous relationships to their corresponding *Prunus *chromosomes. It also suggests that both of the two sub-genomic regions generated by WGD have retained a similar level of conservation of orthology. When the *Malus *chromosomes were divided into sub-genome 1 and 2 prior to the analyses (see Materials and Methods) so that Mercator could find ORs in each *Malus *subgenome, 706 ORs were detected (Table 1). The whole genome duplication of *Malus *alone however does not account for the higher number of rearrangements that occurred since *Prunus *and *Malus *diverged from a common ancestor. Since the ancestor of the genus *Fragaria *diverged from a common ancestor shared by both *Malus *and *Prunus*, it is more likely that there have been more instances of large-scale chromosomal fission in the *Malus *lineage than the occurrence of multiple, yet independent fusion events in the *Prunus *and *Fragaria *lineages to derive the extant genome structure that is evident in the three genera today. More instances of large-scale chromosomal fission may be a consequence of, or related to, the WGD that occurred in *Malus *lineage. Some of the rearrangements, however, may have resulted from the potential errors during genome sequencing and assembly.

### Evaluation of orthologous regions between Fragaria, Malus and Prunus

The evolutionary relationships among the three Rosaceous species studied were analysed further by investigating ORs shared amongst all three genera in addition to those detected in each taxon pair. In total 1399 regions that were orthologous in all three genera were identified. The list of ORs with their positions and orientations in each genome are given in Table S1. Table S2 lists the size of ORs and the number of exons in each genome. The ORs contained 667 out of 855 RosCOS that have been anchored to the peach genome and 616 of the total 1399 ORs contained anchored RosCOS markers. The list of RosCOS markers, their anchored positions and their matching ORs are provided in Table S3. The longest OR in *Prunus *and *Fragaria *was OR 627 spanning 3.5 Mb in PC8 and 1.3 Mb in FC2 with an OR in MC9. The longest OR in *Malus *was 2.6 Mb in MC4 with ORs in PC6 and FC6 (Table 1). OR 627 contained 1318 exons and 316 genes in *Prunus*, 998 exons and 200 genes in *Fragaria*, and 92 exons and 21 genes in *Malus*, respectively. The numbers of sequences in OR 627 with matches in other genomes were 125 exons and 62 genes in *Prunus*, 121 exons and 57 genes in *Fragaria*, and 21 exons and 6 genes in *Malus*, respectively. Table S4 lists all the genes and exons in OR 627 in each genome with their positions. The longest ORs in each genome and size distributions of the ORs are given in Table S5.

**Figure 2:**
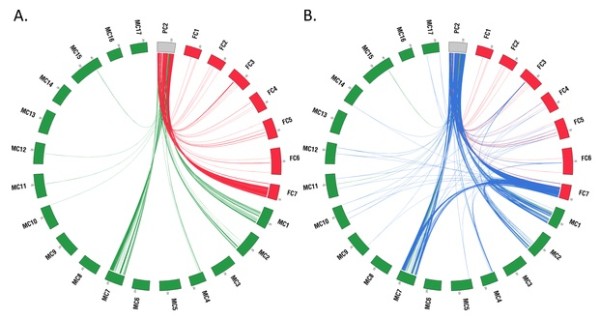
**Comparison of orthologous regions (OR) from two-species analyses and those from the three-species analysis**. A. ORs between PC2 and chromosomes of *Fragaria *and *Malus*, detected from two separate analyses. B. The same ORs shown in A as well as ORs that are shared by all three species. Blue lines link the ORs shared by all three species, red lines link ORs between *Prunus *and *Fragaria *only, and green lines link ORs between *Prunus *and *Malus *only. Data were plotted using Circos [42].

When multiple species are used, as in this analysis, pairwise homology maps can be utilized to build orthology maps for multiple species, as Mercator will find orthologous segments even if some anchors are missing in one of the species. The analysis thus resulted in the detection of additional orthologous regions that were not detected when the taxon pairs were investigated separately (Table 1). The comparison of ORs from the two-species analyses and the comparison of ORs from the three-species analysis are shown in Figure 2. Figure 2A shows ORs between PC2 and chromosomes of *Fragaria *and *Malus*, detected by separate taxon pair analyses. Figure 2B shows the same ORs shown in Figure 2A as well as the ORs shared between all three species. Blue lines link the ORs shared by all three species, red lines link ORs between *Prunus *and *Fragaria *only, and green lines link ORs between *Prunus *and *Malus *only. The figures showing ORs in the other seven *Prunus *chromosomes are shown in Additional file 1: Figure S1. The presence of red lines and green lines in Figure 2B shows that some ORs remain syntenic only between two species, as expected. The comparison of Figure 2A, B also shows additional ORs, which were not detected by the analyses of single taxon pairs. Most notable were the large numbers of additional ORs between *Prunus *and *Malus *that were detected in the three-species analysis. The additional ORs that were detected mostly resided in chromosomes that did not display major orthologous relationships with chromosome PC2 (Figure 2B, Table 2). This result suggests that content and/or order of the genes in ORs that reside on non-orthologous chromosomes went through more rearrangements than those in highly orthologous regions, masking their ancestral origins.

### Comparison of orthologous regions in major orthologous and non-orthologous chromosomes

Further characterization and comparison of ORs in orthologous and non-orthologous chromosomes was performed through an examination of the size and the syntenic quality of the ORs that were conserved in all three species. Syntenic quality was defined as twice the number of matching exons divided by the total number of exons in both segments. The percentage identity (PID) and the bit score of the BLAT matches were also compared. Table 3 shows that the syntenic quality is higher in ORs between major orthologous chromosomes of *Prunus *and *Malus *(21.8%) than those between non-orthologous chromosomes (16.8%). The ORs from both groups however, had similar PIDs and bit scores between BLAT matches. We did not observe many differences in syntenic quality, PID and bit scores between major orthologous and non-orthologous regions in the analysis between the *Prunus *and *Fragaria *genomes, suggesting that chromosomal regions transposed by interchromosomal rearrangements in *Malus *have gone through more changes in terms of gene content and/or gene order, but not in terms of gene sequences. A WGD event followed by massive gene loss, neofunctionalization of genes and other chromosomal changes have been observed in the evolutionary history of extant lineages, including yeast, plant and vertebrates [26-31]. The differences observed may be a consequence of the fact that the *Malus *genome has gone through a recent WGD and as a result has the highest number of predicted genes of any genome sequenced to date [21]. Thus *Malus *may have a greater degree of flexibility in the level of change in gene content and/or gene order that its genome can permit due to two copies of each gene being present than could be tolerated within the *Fragaria *genome., The syntenic quality between the two taxon pairs, however, was similar: 23.6% and 21.1% for *Prunus*/*Fragaria *and *Prunus*/*Malus*, respectively (Table 3).

**Table 3:** Comparisons of orthologous regions (ORs) in major orthologous chromosomes with those in non-orthologous chromosomes

ORs in	No. OR	Mean length in kb (*Prunus*| *Fragaria*)	Mean No. Exons (*Prunus*| *Fragaria*)	Mean No. Matching Exons	Mean Syntenic Quality (%)	Mean PID (%)	Mean Bit Score
**Orthologous chromosomes between *Prunus *and *Fragaria***	1261	151.0|137.3	110|386	27	23.6	87.1	137.3

**non-orthologous chromosomes**	138	134.7|99.1	90|86	23	24.3	87.5	134.3

**All chromosome**	1399	149.4|133.5	108|356	27	23.6	87.1	137.1

**ORs in**	**No. OR**	**Mean length in kb (*Prunus*| *Malus*)**	**Mean No. Exons (*Prunus*| *Malus*)**	**Mean No. Matching Exons**	**Mean Syntenic Quality (%)**	**Mean PID (%)**	**Mean Bit Score**

**Orthologous chromosomes between *Prunus *and *Malus***	1181	133.4|87.6	103|52	26	21.8	89.6	143.3

**non-orthologous chromosomes**	218	236.0|54.6	136|35	29	16.8	90.0	139.7

**All chromosome**	1399	149.4|82.4	108|49	27	21.0	89.7	142.8

Major orthologous chromosomes between *Prunus *and *Fragaria*/*Malus *are listed in Table 2. Regions that are conserved in all three genomes are considered in this comparison

### Detection of conserved ancestral regions

**Figure 3:**
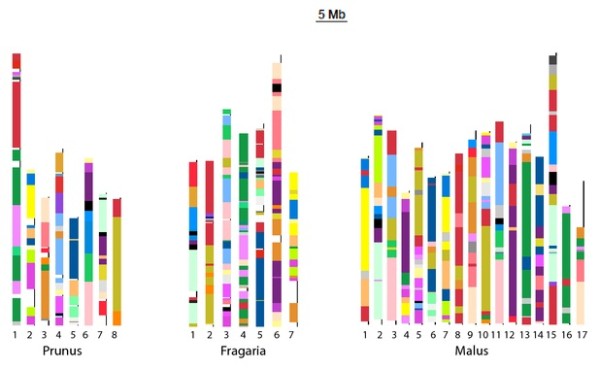
**The chromosomes of *Prunus*, *Fragaria*, and *Malus*, with the colors represent the origin from the 49 contiguous ancestral regions (CARs)**. The spaces with a black line represent chromosomal regions where the ancestral origin was not assigned. CARs that existed before the split of *Prunus*, *Fragaria *and *Malus*, were detected by MGRA (Multiple Genome Rearrangments and Ancestors) algorithm [32]. The figure was drawn using R program (Hornik 2011).

Reconstruction of a hypothetical ancestral genome for Rosaceae was performed using the MGRA (Multiple Genome Rearrangements and Ancestors) algorithm [32]. The *Prunus *and *Fragaria *genomes were used in the analysis with the *Vitis *genome as an outgroup. The *Malus *genome was not included in the MGRA analysis due to the fact that the primary assembly of apple did not include all the predicted genes sequenced. MGRA did not predict the number of chromosomes the ancestral genome contained, but it identified 49 CARs (Contiguous Ancestral Regions) that existed before the divergence of the *Prunus*, *Fragaria *and *Malus *genomes from a common ancestor. Each CAR represents a chromosomal region of the genome of the common ancestor of *Prunus *and *Fragaria*. The ancestral origins of the extant *Malus *chromosomes were inferred through a comparison of corresponding ORs in the *Malus *and *Prunus *genomes. Figure 3 shows the chromosomes of *Prunus*, *Fragaria*, and *Malus*, in which the 49 CARs are depicted in different colors. The results show that chromosomes of *Fragaria *are composed of many small chromosomal regions that originated from different ancestral CARs compared to those of *Malus *and *Prunus *(Figure 3), suggesting that the *Fragaria *genome went through a greater number of small scale rearrangements compared to the genomes of the other genera since they diverged from a common ancestor (Figure 3). Table 4 shows that the number of breaks between the chromosomal regions originating from different CARs in *Fragaria *is over two times greater than that in *Malus *and over 1.5 times greater than that in *Prunus*. The genomes of the diploid and the octoploid *Fragaria *that have been investigated to date through comparative mapping have been shown to be largely collinear [33,34], however, whether the occurrence of small chromosomal rearrangements is common in the entire *Fragaria *lineage or restricted to species closely related to *F. vesca *would require further investigation.

**Table 4:** Number of breaks between chromosomal regions that are originated from different CARs

*Malus*		*Prunus*		*Fragaria*	
**chromosome**	**No. break**	**chromosome**	**No. break**	**chromosome**	**No. break**

1	5	scaffold_1	26	LG1	14

2	12	scaffold_2	11	LG2	9

3	8	scaffold_3	6	LG3	12

4	8	scaffold_4	15	LG4	37

5	15	scaffold_5	8	LG5	25

6	8	scaffold_6	8	LG6	15

7	10	scaffold_7	12	LG7	15

8	6	scaffold_8	5		

9	9				

10	13				

11	9				

12	6				

13	13				

14	9				

15	14				

16	7				

17	6				

**Sum**	158.0		91.0		127.0

**Avg. (per 10 Mbp)**	3.0		4.2		6.4

**Avg. (per chromosome)**	9.3		11.4		18.1

### Reconstruction of hypothetical Rosaceae ancestral genome

**Figure 4:**
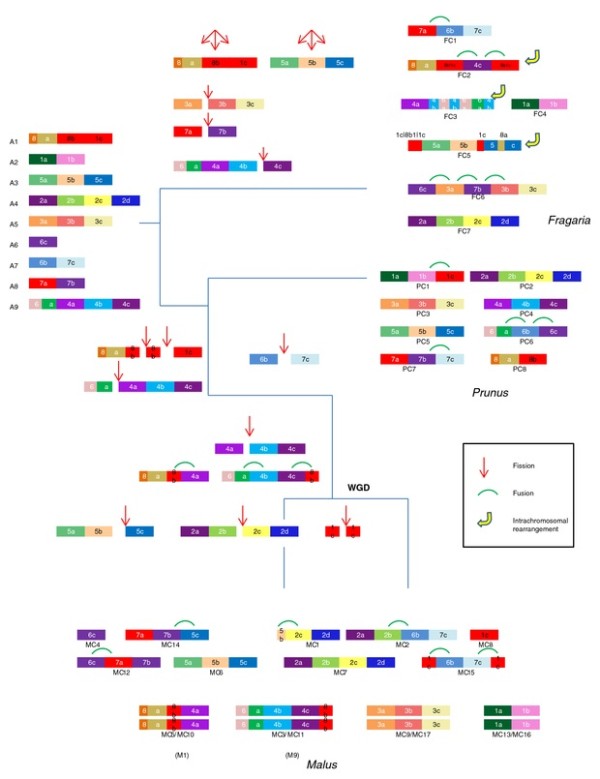
**Hypothetical evolutionary steps from the nine Rosaceae ancestral chromosomes to *Fragaria*, *Prunus *and *Malus *lineage**. Each color represent distinct CARs detected by MGRA algorithm. Chromosomal rearrangements specific for Rosoideae (contains *Fragaria*) and Spireaoideae (contains *Malus *and *Prunus*) are depicted. Also shown are chromosomal rearragenments specific for *Prunus*, *Malus*, and subgenome of *Malus *after the WGD.

Since the genus *Fragaria *split from the common ancestor of *Malus *and *Prunus *before those species diverged, if regions with the same ancestral origin reside in the same chromosome of both *Prunus *and *Fragaria*, but in different chromosomes of *Malus*, we can infer that the those chromosomes of *Malus *were generated by a fission event. Likewise, if regions with the same ancestral origin reside in the same chromosome of *Prunus *but in different chromosomes of *Malus *and *Fragaria*, we can infer the chromosome of *Prunus *was generated by a fusion event. In this way, we have constructed a hypothetical ancestral karyotype, consisting of nine chromosomes, using the top 24 CARs identified in this investigation (Figure 4). The orthology maps between the three species, which support the hypothesis, are shown in Additional file 2: Figure S2. Figure 4 shows that the *Fragaria *lineage went through at least five fission events and seven fusion events, not including intrachromosomal rearrangements, the *Prunus *lineage went through at least three fission events and four fusion events and the *Malus *lineage went through seven fission events and nine fusion events. Two fission events occurred after the split of *Fragaria *and before the split of *Malus *and *Prunus*. Two further fission events and three fusion events occurred before the WGD of *Malus *lineage and the three further fission events occurred after the WGD in only one of the two homeologous chromosomes (Figure 4) of *Malus*. These data suggest that the *Prunus *lineage has the most conserved karyotype of the three species investigated and that the *Malus *lineage went through the most large-scale chromosomal fission/fusion events. It is also clear that intrachromosomal genome rearrangements played an important role in the genome evolution of the genus *Fragaria*. Additionally, Figure 4 suggests that the karyotypes of the ancestor of *Malus *existed before the WGD, as M1, M9 and A2 to A8. M1 and M9 were generated from A1 and A9, after four fissions and three fusions, and correspond to the present *Malus *chromosomes MC5/MC10 and MC3/MC11, respectively. Our result is consistent with previous phylogenetic analyses [21,35] and the analysis of comparative mapping data [2], in suggesting that both the ancestors of Rosaceae and *Malus *have genomes consisting of nine chromosomes.

**Figure 5:**
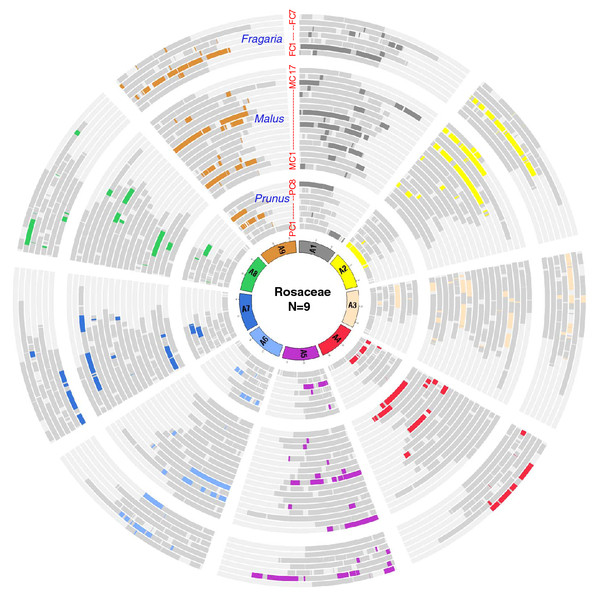
**The Concentric circle of Rosaceae genomes**. The innermost circle represents the putative nine chromosomes of Rosaceae ancestral genome. Next sets of circles represent eight, 17 and seven chromosomes of *Prunus*, *Malus *and *Fragaria*, respectively. The regions originated from each Rosaceae ancestral chromosome are highlighted with corresponding color in Figure S3. The Data were plotted using Circos [42].

To show how the genomes of the three taxa have evolved since they diverged from this common ancestral karyotypes, the nine ancestral chromosomes, A1 through A9, along with genomes of three species, colored by the ancestral chromosomal origin, were constructed (Additional file 4: Figure S3). In this figure, the 24 CARs in Figure 4 were reassigned with colors based on which of the nine ancestral chromosomes they reside in. The orthologous relationships amongst the three Rosaceae genomes are shown in the Rosaceae concentric circle with the putative nine chromosomes of Rosaceae ancestral genome as the innermost circle (Figure 5). This allows the identification of orthologous regions between the three genomes that have a common ancestral origin.

## Conclusions

The availability of whole genome sequence data has permitted for the first time a detailed evaluation of the conservation of macro- and micro-synteny in the Rosaceae which has demonstrated that the genomes of *Fragaria*, *Malus *and *Prunus *have undergone different modes of evolution since they diverged from a common ancestor. This study has revealed that a greater number of small scale rearrangements have occurred in *Fragaria *than in either *Malus *or *Prunus *and has indicated that *Malus *went through more translocations potentially as a consequence of the WGD event in the lineage of the genus. The results of this investigation suggest that *Prunus *has the most conserved karyotype at both the macro- and micro-syntenic level in relation to the ancestral genome configuration for the Rosaceae, which in concordance with other studies is hypothesised to have had nine chromosomes. The resolution obtained in this comparison of genome structure demonstrates the utility of whole genome sequencing data to the elucidation of mechanisms driving genome evolution between related organisms at a level of resolution that would not have been possible through conventional comparative mapping endeavours.

## Materials and methods

### Detection of orthologous regions

To detect orthologous regions between the peach and grape genomes, the whole genome sequence and annotation data of grape were downloaded from Genoscope [36]. Whole genome sequence of *Prunus persica *v1.0, primary assembly of *Malus domestica *and *Fragaria vesca beta version FvH4 pseudochromosomes *were downloaded from GDR, Genome Database for Rosaceae [37,38]. The annotation data that includes the prediction of exons and genes were also downloaded from the databases above. All the sequence and annotation files that have been used in this study are available from GDR http://www.rosaceae.org/BMC_rosaceae_Genome_paper. The whole genome sequences of peach and grape were masked for repeats using RepeatMasker [39], as well as the nmerge, WU-BLAST distribution, and faSoftMask distribution utilities of Mercator [25]. Mercator identifies orthologous regions with one to one ortholgy relationships, rather than producing any syntenic regions in which one region can have many syntenic regions. Mercator employs BLAT-similar anchor pairs to identify orthologous segments in a modified k-way reciprocal best hit algorithm [40]. Translated sequences of exons, provided by the annotation data, have been used as anchors in these analyses. Two exons from each genome were determined to be similar if the BLAT [41] score of the pair was below 1e -10. BLAT scores were computed in protein space. To select the optimal criteria to assess conservation of synteny between Rosaceous genomes, Mercator parameters were varied from between a minimum of 30 exons and a maximum distance of 300 kbp between exons, to a minimum of two exons and a maximum distance of 3 Mbp between exons. As the parameters become less stringent, we observed a sudden increase of the number of orthologous regions without the accompanying increase of the percent geonome coverage. Parameters selected for further analysis were a minimum of ten exons and a maximum distance of 300 kbp between exons as these parameters gave high percentage coverage within the genomes but reduced small-size syntenic regions that are potentially artefactual. With the exception of the analysis shown in Figure 1, the *Malus *genome was split into two arbitrary 'sub-genomes' based on the data of Velasco et al [21]; sub-genome 1 consisted of chromosomes 1, 2, 3, 4, 5, 8, 9, 13 and 14, whilst sub-genome 2 was composed of chromosomes 6, 7, 10, 11, 12, 15, 16 and 17 to use as an input for the Mercator program. This was done to detect orthologous regions in each of the homeologous *Malus *chromosomes. The anchored position of RosCOS markers in the peach genome were downloaded from GDR [37,38]. RosCOS markers were anchored to orthologous regions when their anchored positions in peach belong to the corresponding positions of ORs.

### Reconstruction of hypothetical ancestral genome

We used the Multiple Genome Rearrangements and Ancestors (MGRA) algorithm [32] to predict Contiguous Ancestral Regions (CARs) that existed in a common ancestor. The orthology map of *Prunus*, *Fragaria *and *Vitis *genomes, produced by Mercator, was used as an input for the MGRA program. The *Vitis *genome was included in the analysis as anoutgroup. The hypothetical ancestral genome was manually constructed using CARs generated from MGRA, as written in the Result and discussion section above.

## Abbreviations

CARs: Contiguous ancestral regions; MGRA: Multiple genome rearrangements and ancestors; OR: Orthologous region; PID: Percentage identity; RosCOS: Rosaceous conserved orthologous sequences; SSR: Simple sequence repeat; WGD: Whole genome duplication.

## Competing interests

The authors declare that they have no competing interests.

## Authors' contributions

SJ designed the study, performed the analysis, analyzed the data and wrote the paper. AC and MT participated in the design of the study, analyzed the data and critically revised the manuscript. DM participated in the design of the study and critically revised manuscript. PZ made figures that show contiguous ancestral regions using R program. IC wrote scripts for parsing data from Mercator output. KF, BS, AA, JMC, PA, VS, MM, DR, IV and RV conceived of the study and critically revised the manuscript. DS participated in the design of the study, analyzed the data and participated in writing. All authors read and approved the final manuscript.

## Supplementary Material

Additional file 3 **Table S1: List of ORs that are conserved in all three genomes with their positions and orientations in each game**. Click here for file

Additional file 1 **Figure S1**. Comparison of orthologous regions (OR) from two-species analysis and those from the three-species analysis. ORs between a *Prunus *chromosome (A:PC1, B:PC3, C:PC4, D:PC5, E:PC6, F:PC7, G:PC8) and chromosomes of *Fragaria *and *Malus*, detected from two separate analyses are shown in the diagram on the left. The same ORs shown in the diagram on the left as well as ORs that are shared by all three species are shown in the diagram on the right. Blue lines link the ORs shared by all three species, red lines link ORs between *Prunus *and *Fragaria *only, and green lines link ORs between *Prunus *and *Malus *only. Data with PC2 is shown in Figure 2 of the main manuscript. Data were plotted using Circos (Krzywinski et al. 2009). Click here for file

Additional file 2 **Figure S2**. Orthology map identified between *Prunus *and the other two Rosaceous genera based on whole genome sequence analysis. The lines link one to one orthologous region identified using Mercator program (Dewey 2007). Only the orthologous regions between the major orthologous chromosomes, as shown in Table 2, are depicted. The colors represent the contiguous ancestral regions (CARs). The spaces with a black line represent chromosomal regions where the ancestral origin was not assigned. CARs that existed before the split of *Prunus*, *Fragaria *and *Malus*, were detected by MGRA (Multiple Genome Rearrangments and Ancestors) algorithm (Alekseyev and Pevzner 2009). A through H shows orthologous regions in *Fragaria *and *Malus *corresponding to those in *Prunus *chromosome 1 through 8, respectively. Click here for file

Additional file 4 **Figure S3**. The chromosomes of *Prunus*, *Fragaria*, and *Malus*, with the colors represent the origin from the nine putative chromosomes of Rosaceae ancestor. The spaces with a black line represent chromosomal regions where the ancestral origin was not assigned. For this figure, the top 24 CARs in Figure 4 were assigned to a distinct color, depending on which of the nine chromosomes of Rosaceae ancestor they belong to. The figure was drawn using R program (Hornik 2011). Click here for file
