# Supplementary material for: Whole genome comparisons of Fragaria, Prunus and Malus reveal different modes of evolution between Rosaceous subfamilies
Source: BMC Genomics. 2012 Apr 4;13:129. doi: 10.1186/1471-2164-13-129 (PMC3368713; doi:10.1186/1471-2164-13-129)
Supplement: Additional file 1 — Figure S1. Comparison of orthologous regions (OR) from two-species analysis and those from the three-species analysis. ORs between a Prunus chromosome (A:PC1, B:PC3, C:PC4, D:PC5, E:PC6, F:PC7, G:PC8) and chromosomes of Fragaria and Malus, detected from two separate analyses are shown in the diagram on the left. The same ORs shown in the diagram on the left as well as ORs that are shared by all three species are shown in the diagram on the right. Blue lines link the ORs shared by all three species, red lines link ORs between Prunus and Fragaria only, and green lines link ORs between Prunus and Malus only. Data with PC2 is shown in Figure 2 of the main manuscript. Data were plotted using Circos (Krzywinski et al. 2009). [file 1471-2164-13-129-S1.PPT]

## Slide 1
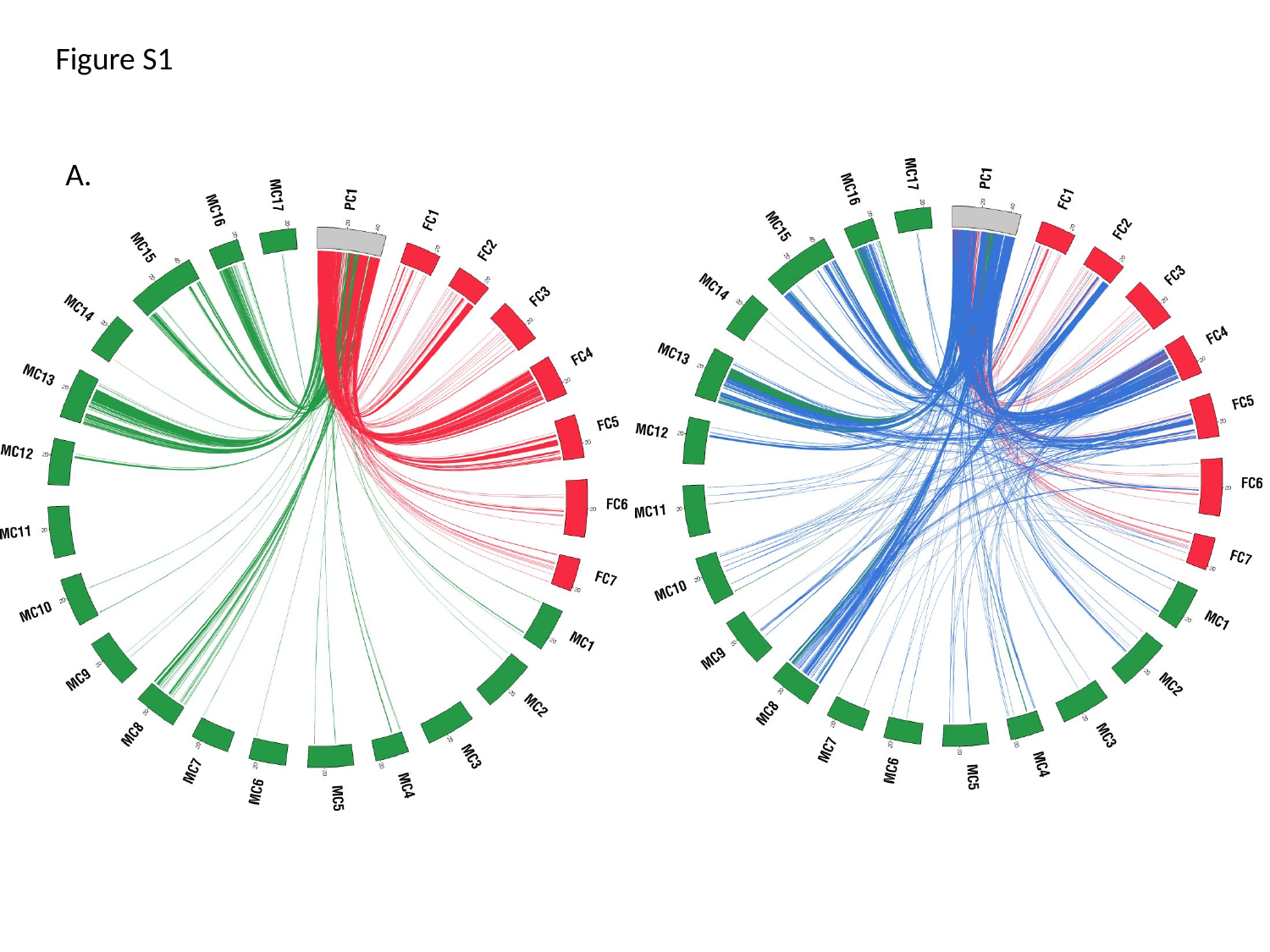

Figure S1
A.

## Slide 2
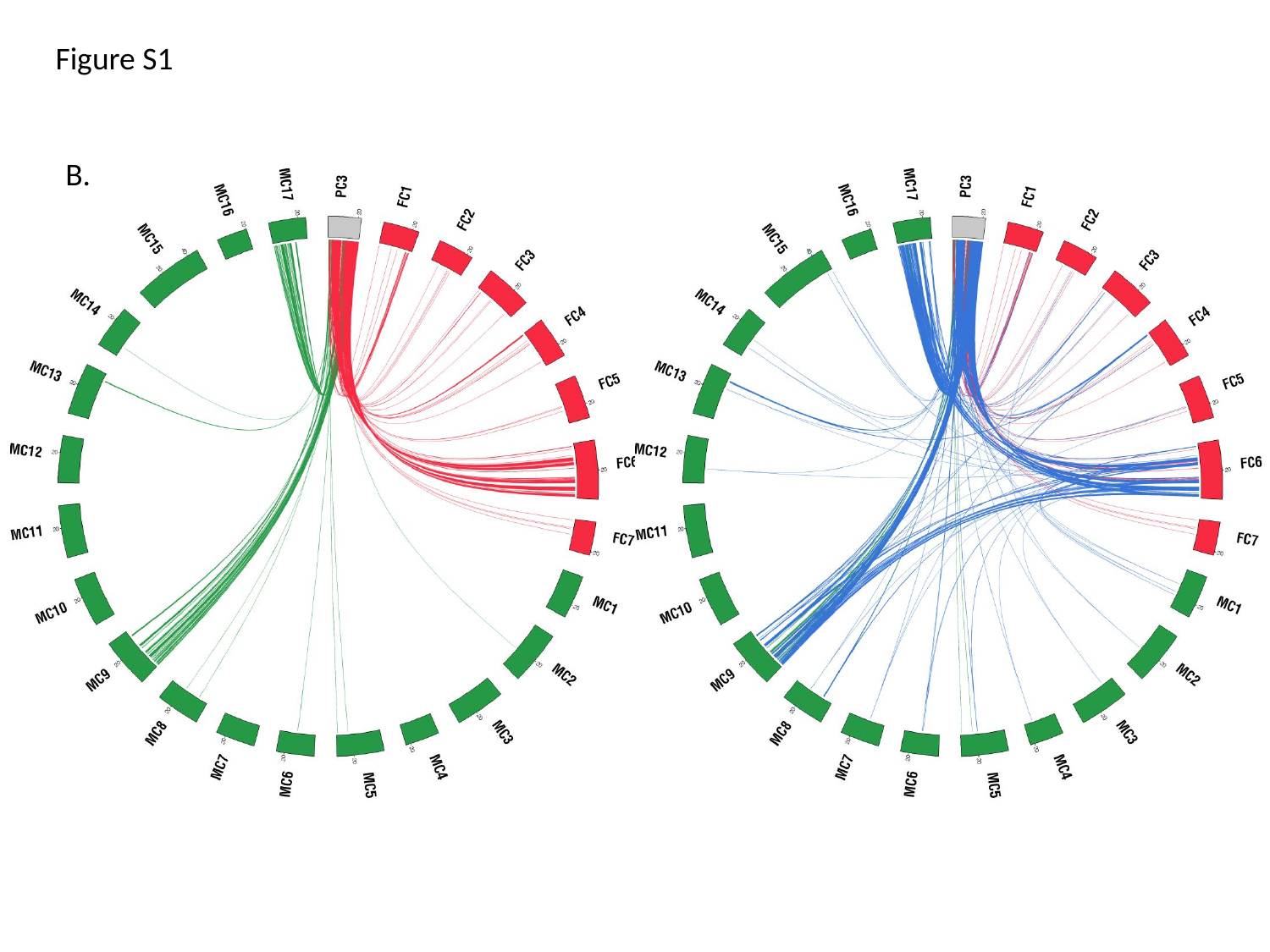

Figure S1
B.

## Slide 3
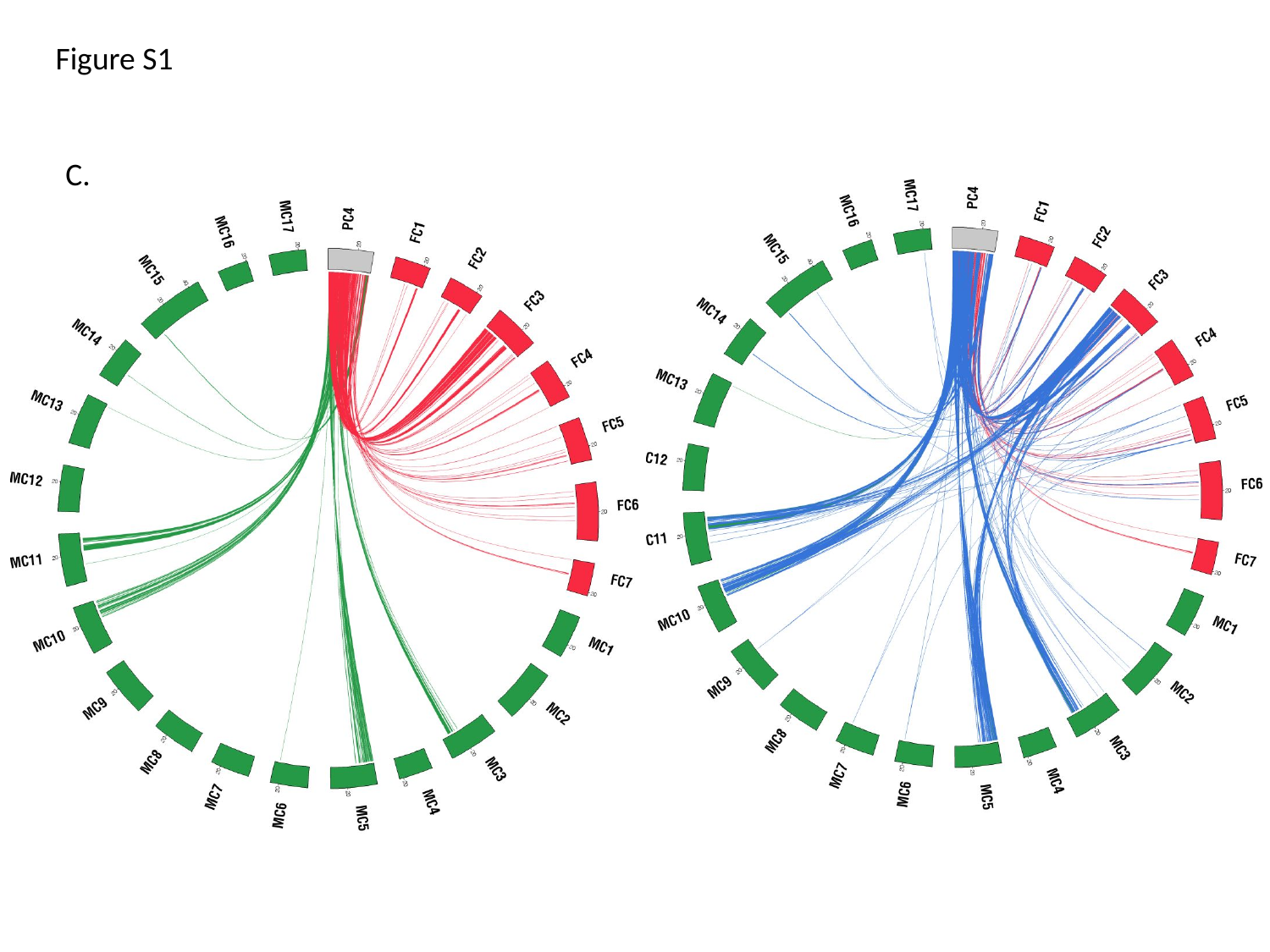

Figure S1
C.

## Slide 4
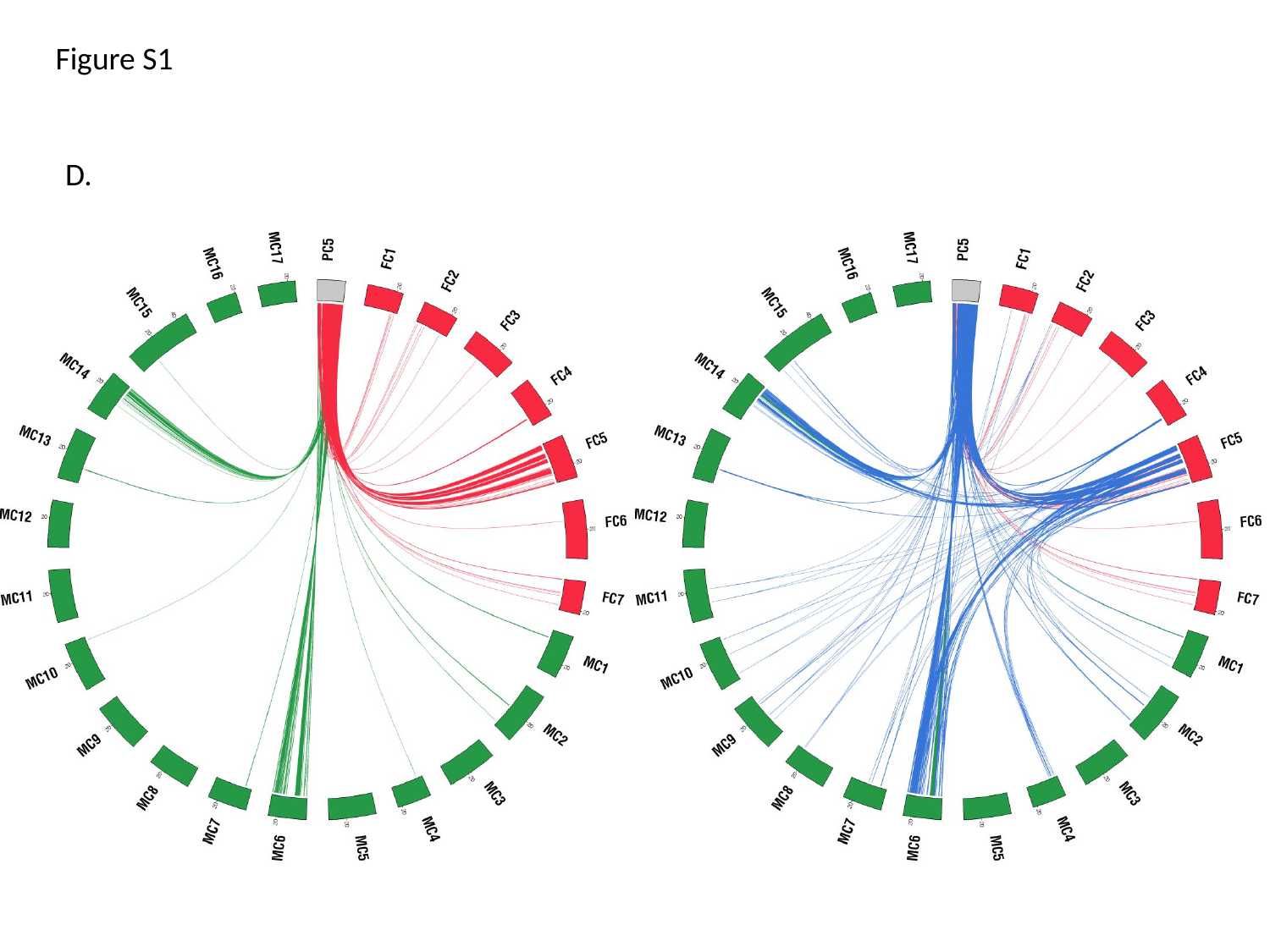

Figure S1
D.

## Slide 5
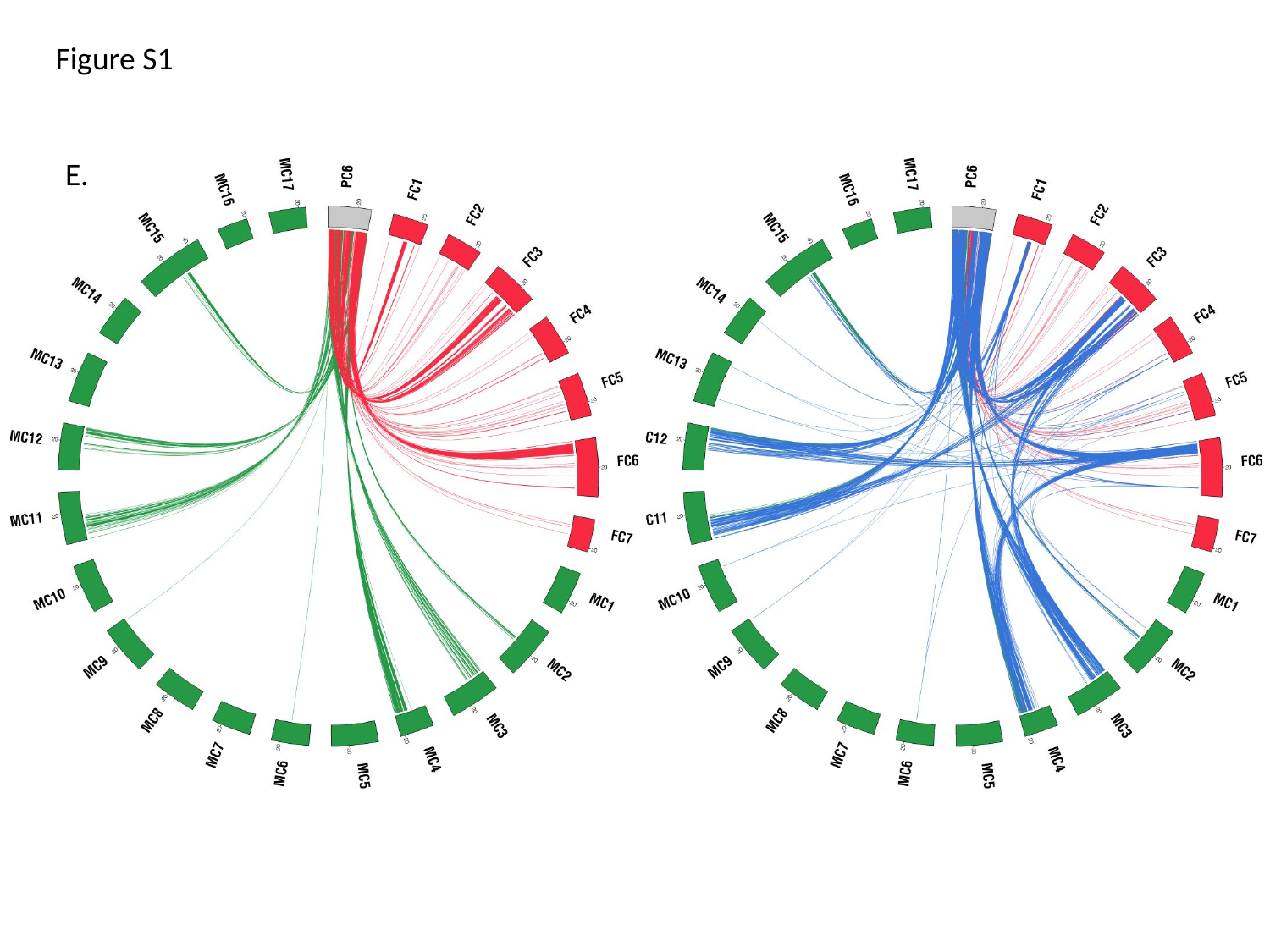

Figure S1
E.

## Slide 6
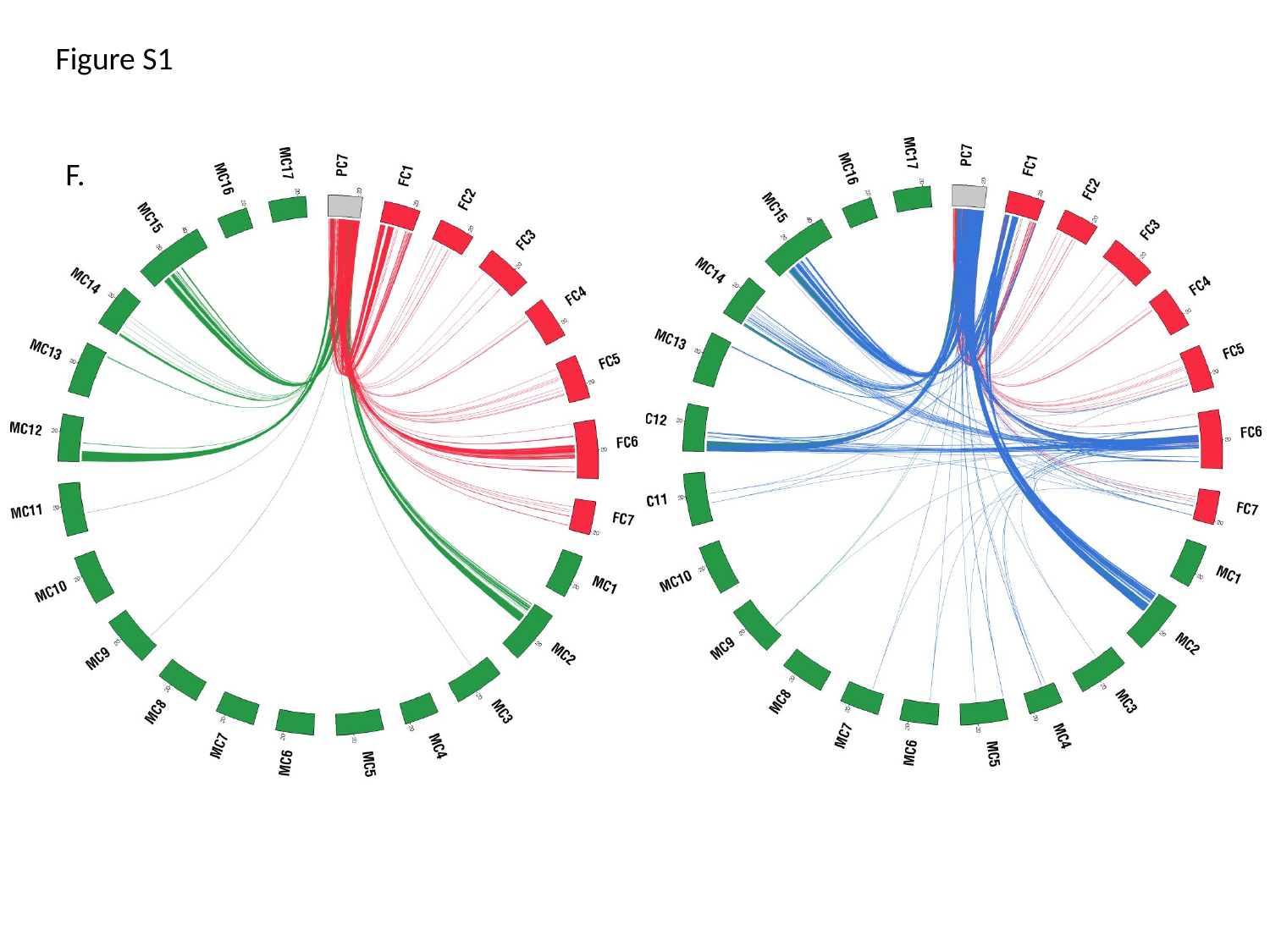

Figure S1
F.

## Slide 7
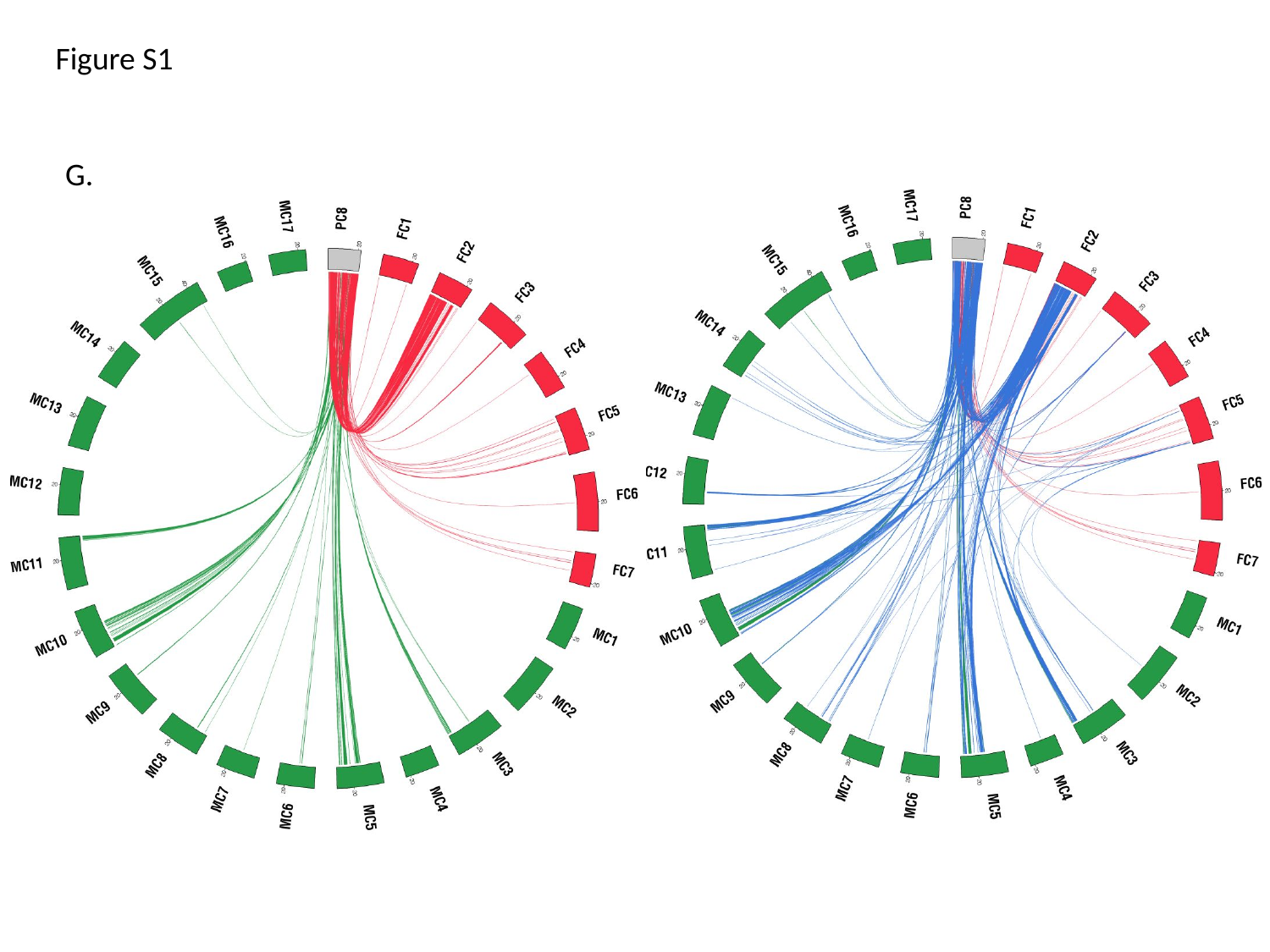

Figure S1
G.
