# Supplementary figures and images for: Whole genome comparisons of Fragaria, Prunus and Malus reveal different modes of evolution between Rosaceous subfamilies
Source: BMC Genomics. 2012 Apr 4;13:129. doi: 10.1186/1471-2164-13-129 (PMC3368713; doi:10.1186/1471-2164-13-129)

## Slide 1
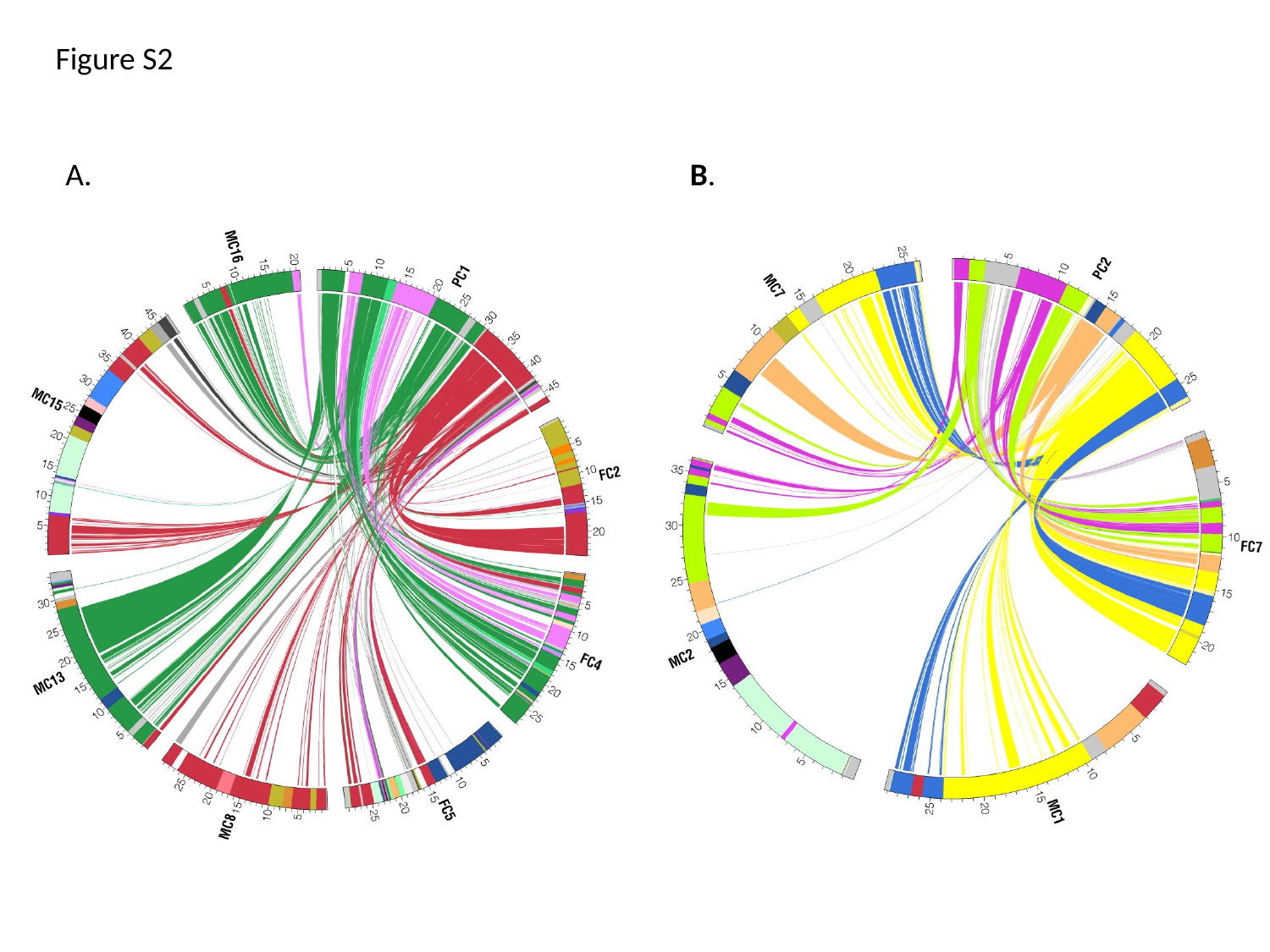

Figure S2
A.
B.

## Slide 2
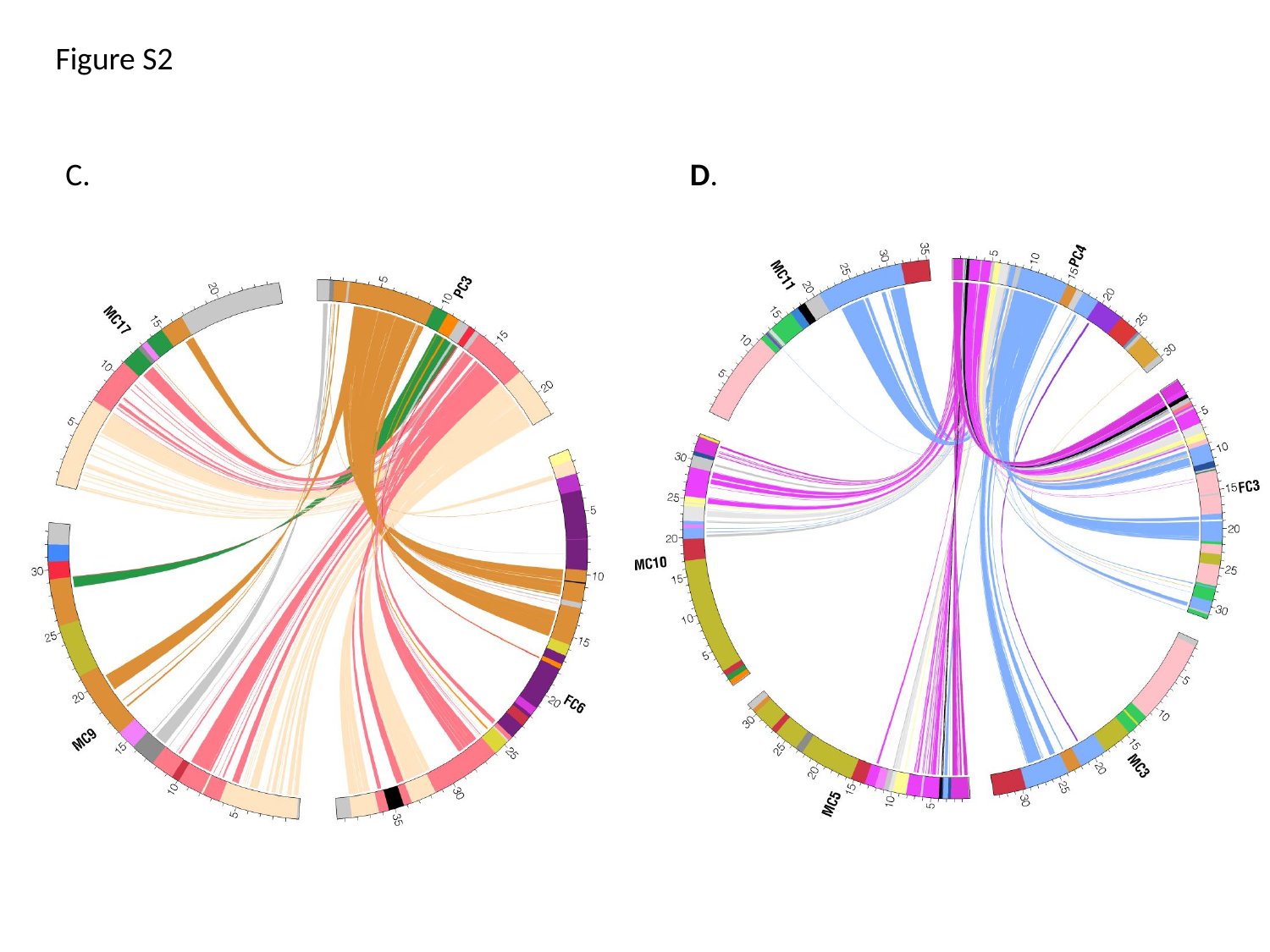

Figure S2
C.
D.

## Slide 3
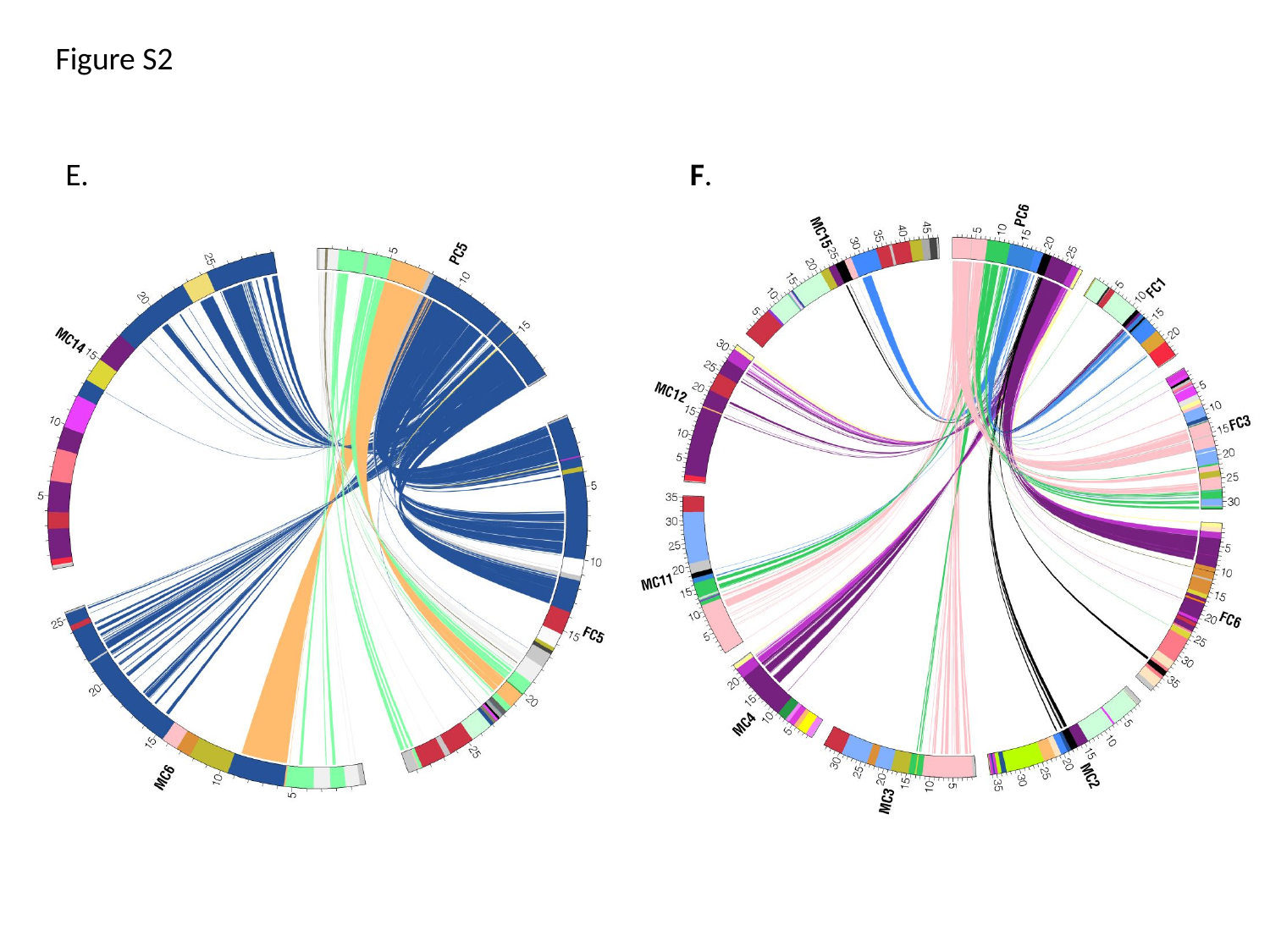

Figure S2
E.
F.

## Slide 4
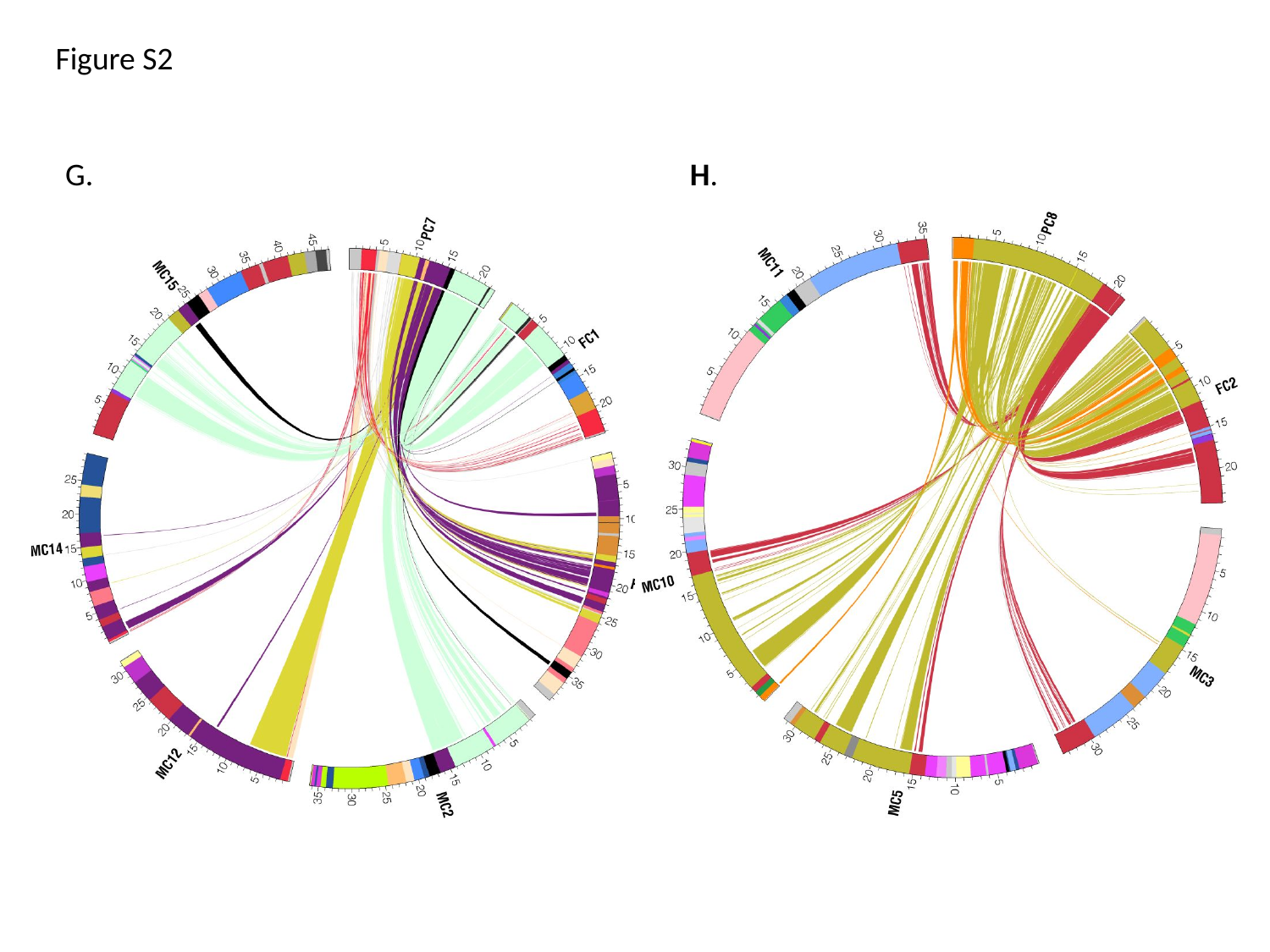

Figure S2
G.
H.

Supplement: Additional file 2 — Figure S2. Orthology map identified between Prunus and the other two Rosaceous genera based on whole genome sequence analysis. The lines link one to one orthologous region identified using Mercator program (Dewey 2007). Only the orthologous regions between the major orthologous chromosomes, as shown in Table 2, are depicted. The colors represent the contiguous ancestral regions (CARs). The spaces with a black line represent chromosomal regions where the ancestral origin was not assigned. CARs that existed before the split of Prunus, Fragaria and Malus, were detected by MGRA (Multiple Genome Rearrangments and Ancestors) algorithm (Alekseyev and Pevzner 2009). A through H shows orthologous regions in Fragaria and Malus corresponding to those in Prunus chromosome 1 through 8, respectively. [file 1471-2164-13-129-S2.PPT]

## Slide 1
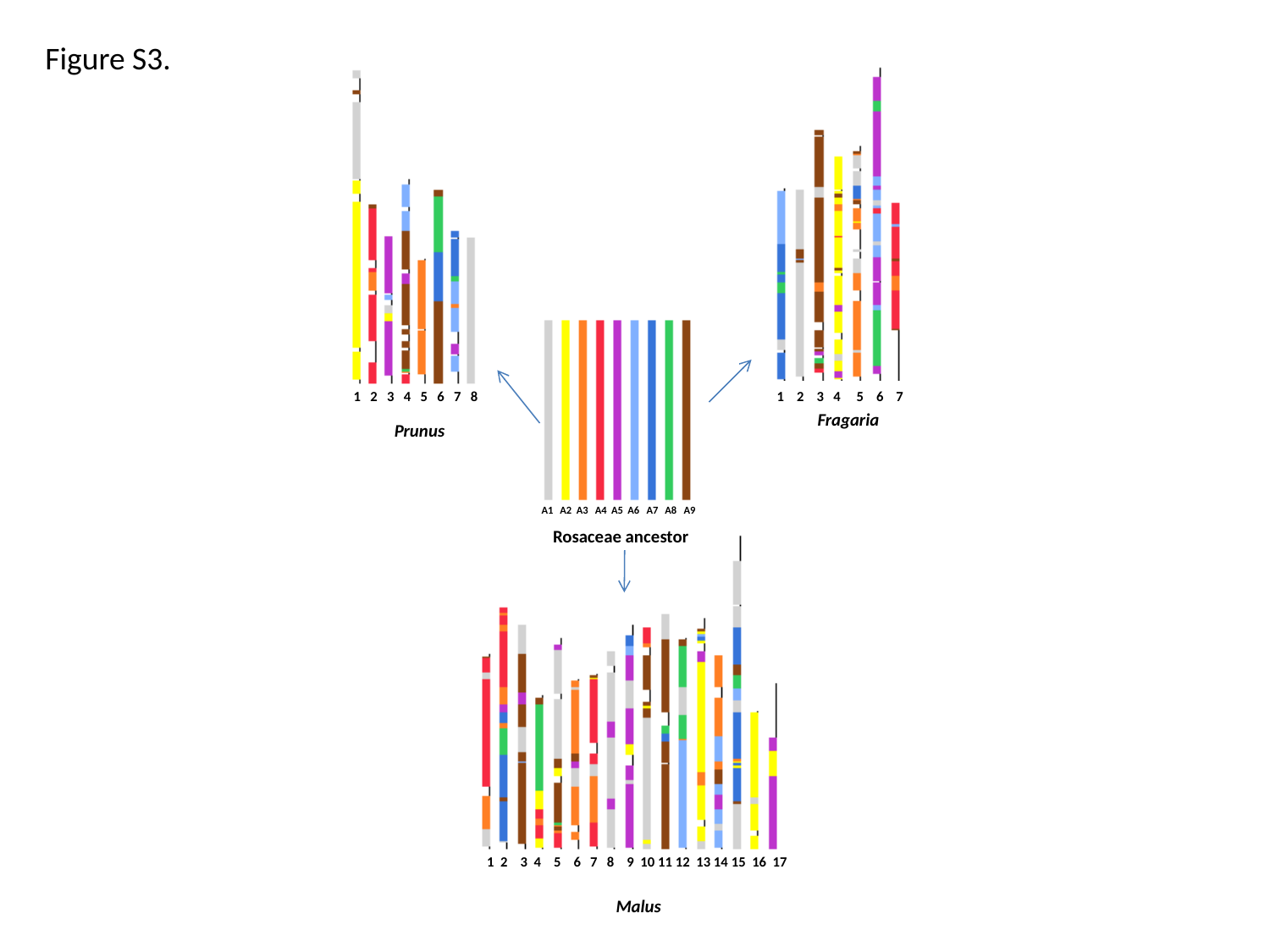

Figure S3.
 1 2 3 4 5 6 7 8
 1 2 3 4 5 6 7
Fragaria
Prunus
A1 A2 A3 A4 A5 A6 A7 A8 A9
Rosaceae ancestor
1 2 3 4 5 6 7 8 9 10 11 12 13 14 15 16 17
Malus

Supplement: Additional file 4 — Figure S3. The chromosomes of Prunus, Fragaria, and Malus, with the colors represent the origin from the nine putative chromosomes of Rosaceae ancestor. The spaces with a black line represent chromosomal regions where the ancestral origin was not assigned. For this figure, the top 24 CARs in Figure 4 were assigned to a distinct color, depending on which of the nine chromosomes of Rosaceae ancestor they belong to. The figure was drawn using R program (Hornik 2011). [file 1471-2164-13-129-S4.PPT]
